# Subregion specific neuroadaptations in the female rat striatum during acute and protracted withdrawal from nicotine

**DOI:** 10.1007/s00702-023-02678-7

**Published:** 2023-07-27

**Authors:** Oona Lagström, Edvin Vestin, Bo Söderpalm, Mia Ericson, Louise Adermark

**Affiliations:** 1 Department of Pharmacology, Institute of Neuroscience and Physiology, The Sahlgrenska Academy, University of Gothenburg, Gothenburg, Sweden; 2 Addiction Biology Unit, Department of Psychiatry and Neurochemistry, Institute of Neuroscience and Physiology, The Sahlgrenska Academy, University of Gothenburg, Gothenburg, Sweden; 3 Beroendekliniken, Sahlgrenska University Hospital, Gothenburg, Sweden

**Keywords:** Nicotine, Nucleus accumbens, Electrophysiology, Elevated plus maze, Withdrawal

## Abstract

Epidemiological studies and clinical observations suggest that nicotine, a major contributor of the global burden of disease, acts in a partially sex specific manner. Still, preclinical research has primarily been conducted in males. More research is thus required to define the effects displayed by nicotine on the female brain. To this end, female rats received 15 injections of either nicotine (0.36mg/kg) or saline, over a 3-week period and were then followed for up to 3 months. Behavioral effects of nicotine were assessed using locomotor activity measurements and elevated plus maze, while neurophysiological changes were monitored using ex vivo electrophysiological field potential recordings conducted in subregions of the dorsal and ventral striatum. Behavioral assessments demonstrated a robust sensitization to the locomotor stimulatory properties of nicotine, but monitored behaviors on the elevated plus maze were not affected during acute (24 h) or protracted (3 months) withdrawal. Electrophysiological recordings revealed a selective increase in excitatory neurotransmission in the nucleus accumbens shell and dorsomedial striatum during acute withdrawal. Importantly, accumbal neuroadaptations in nicotine-treated rats correlated with locomotor behavior, supporting a role for the nucleus accumbens in behavioral sensitization. While no sustained neuroadaptations were observed following 3 months withdrawal, there was an overall trend towards reduced inhibitory tone. Together, these findings suggest that nicotine produces selective transformations of striatal brain circuits that may drive specific behaviors associated with nicotine exposure. Furthermore, our observations suggest that sex-specificity should be considered when evaluating long-term effects by nicotine on the brain.

## Introduction

Nicotine, the active ingredient in tobacco products, is a highly addictive substance and tobacco use causes 8 million deaths per year worldwide (Global Burden of Disease, ﻿201﻿9). Smoking increases the risk for both cardiovascular and lung diseases as well as cancer, and in women the mortality rate of lung cancer measures higher than that of breast cancer in several countries (World Health Organization, 2020). Available smoking cessation treatments have limited effects and most who try to quit relapse (Livingstone-Banks et al. 2019). Although the health risks of smoking are well known, the prevalence of nicotine dependence is higher than for any other addictive substances worldwide, and the number of women who smoke is still increasing in many regions (Amos and Haglund 2000; Ernster et al. 2000).

Nicotine exposure leads to a dynamic activation and desensitization of nicotinic acetylcholine receptors (nAChRs) and repeated exposure results in a selective upregulation of nAChRs that appears to drive behaviors associated with nicotine reward (Fenster et al. 1999; Picciotto et al. 2008; Peng et al. 1994; Akers et al. 2020). While the reinforcing properties of nicotine especially have been attributed to its effect on neurotransmission in the ventral tegmental area and the concomitant elevation of dopamine levels in the ventral striatum (nucleus accumbens, nAc) (Imperato et al. 1986; Nisell et al. 1994; Pidoplichko et al. 1997; Akers et al. 2020), other striatal regions may also be important for nicotine induced behaviors. In fact, dorsal striatum regions, which are essential for both goal-directed behavior and habit formation, also appear to undergo neuroplastic transformations in response to repeated nicotine exposure (Adermark et al. 2016; Smith and Graybiel 2016; Clemens et al. 2014; Domi et al. 2023a). Changes in dorsal striatal dopaminergic neurotransmission have further been connected to behavioral sensitization to drugs of abuse and compulsive drug-seeking behavior, as well as cue-induced craving and relapse, all critical components for the later stages of addiction (Bamford et al. 2008; Volkow et al. 2006; Durieux et al. 2012). Considering that distinct subregions of the striatum appear to work together to drive nicotine-induced behaviors (Domi et al. 2023a), monitoring parallel neuroplasticity in striatal subregions may be a valuable approach for understanding the neurophysiological underpinnings of nicotine addiction.

Sex-specific differences have been demonstrated in both human and animal studies to all phases of nicotine use, from the initiation of use and through escalation of intake and addiction as well as during abstinence and relapse (Benowitz and Hatsukami 1998; Sanchis-Segura and Becker 2016). Amongst smokers, men are suggested to be more reinforced by nicotine itself, whereas women report more negative effects, more cue-induced craving and more difficulties quitting (Doran 2014; Perkins et al. 2001; Piper et al. 2010). Sex-specific differences in nicotine-induced dopamine release have previously been shown in human studies, and it is suggested that nicotine activates dorsal striatal areas in females, as compared to ventral striatal areas in males (Cosgrove et al. 2014). Preclinical studies reveal a similar pattern, with sex-specific differences indicated in neural circuits involved in motivation and reinforcement as well as stress reactivity and self-control (Carroll and Anker 2010; Lynch et al. 2002). Since both clinical and preclinical studies suggest sex-specific responses related to nicotine consumption, and since most studies have been performed in male subjects, it is of importance to shed light on how nicotine affects the female brain (Carroll and Lynch 2016). The overall aim of this study was thus to investigate behavioral and neurophysiological adaptations elicited by repeated nicotine exposure followed by protracted withdrawal in subregions of the female rat striatum.

## Materials and methods

### Experimental design

**Fig. 1:**
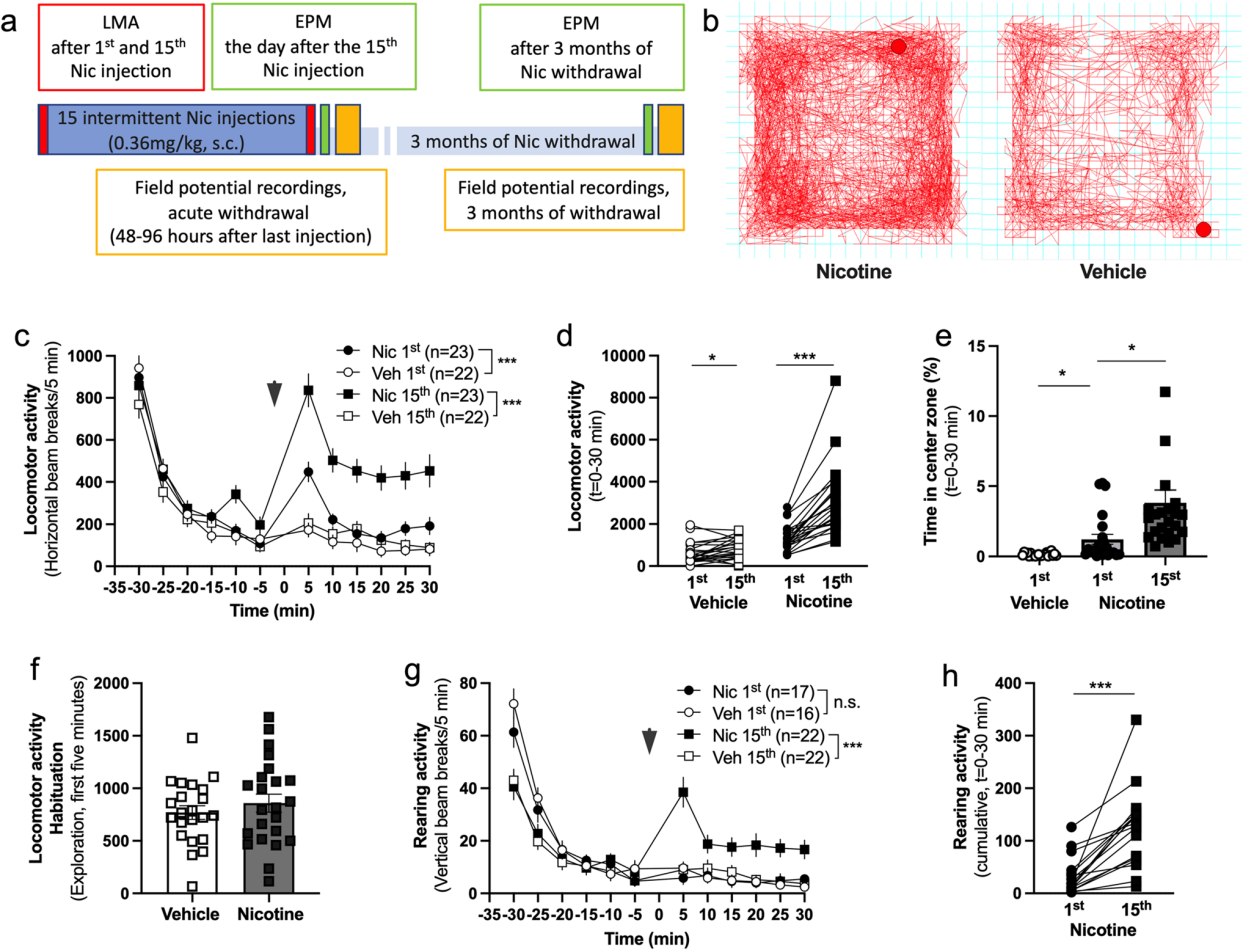
Repeated nicotine administration sensitizes female rats to the locomotor stimulatory properties of nicotine. **a** Schematic graph demonstrating the study outline. **b** Example traces showing movement in the LMA box for a nicotine treated animal and a vehicle treated animal. **c** Time course graph demonstrating locomotion in the LMA box during the first and last injection of either nicotine or saline. Arrow marks the time-point for injection with either nicotine or saline. **d** Increased locomotion was seen following repeated administration of both vehicle and nicotine. **e** Nicotine exposure slightly enhanced the time spent in center zone, and repeated exposure further enhanced the duration. **f** Locomotion during the first five minutes of exploration to the text environment did not differ significantly following repeated nicotine administration. **g** Time-course graph demonstrating rearing activity during habituation and in response to drug administration. Rearing activity was not affected by the first nicotine injection but was notably increased after 15 nicotine injections as compared to vehicle. Arrow marks the timepoint for injection with either nicotine or vehicle. **h** Graph demonstrates cumulative rearing activity in response to the first and last exposure to nicotine in individual rats. Data are presented as mean ± SEM. *n* number of rats. *p < 0.05, **p < 0.01, ***p < 0.001

Female Wistar rats (n = 45) received either nicotine or saline injections over a 3-week period. Locomotor activity (LMA) measurements were performed during the first and last injection and elevated plus-maze (EPM) 24 h after the last injection. Twenty-four rats were used for electrophysiological field potential recordings at the timepoint of 48–96 h of withdrawal, while another group of 18 rats remained in withdrawal for 3-months before EPM and neurophysiological assessments were performed (Fig. 1a).

### Animals

Adult female Wistar rats (Envigo, Netherlands), weighing 180–200 g on arrival, were group housed with a 12-h light/dark cycle (lights on at 7.00 AM), at 21–22 °C and 55% humidity, and ad libitum access to water and chow. Rats were allowed 7 days of habituation to the facilities before starting any experiments. All experiments were approved by the Ethics Committee for animal experiments, Gothenburg, Sweden.

### Nicotine treatment

Female rats were randomly assigned to receive either vehicle (n = 22) or nicotine (n = 23). (−)-Nicotine hydrogen tartrate salt (Merck KGaA, Darmstadt, Germany) was dissolved in 0.9% NaCl with pH adjusted to 7.2–7.4 with NaHCO_3_ and administered subcutaneously at 1 mg/kg (0.36 mg/kg nicotine). Rats received a total of 15 injections over a period of 3 weeks, and the experiments were conducted in four separate batches of animals. This nicotine-injection protocol has previously been shown to induce a robust behavioral sensitization to the locomotor stimulatory properties of nicotine that sustains even after 17 months of withdrawal in male rats (Morud et al. 2016).

### Locomotor activity

Locomotor activity was measured during the first and fifteenth nicotine/saline injection as previously described (Morud et al. 2016). In brief, rats were allowed one hour of habituation to the room in their home cages on the day of the experiments. The rats were placed in the center of a weakly lit locomotor activity (LMA) box (40 × 40 cm, Med Assoc., Fairfax, VT, USA) and allowed an additional 30 min of habituation time in the box, followed by 30 min of measurements after the nicotine/vehicle administration. Ambulatory movement and rearing activity were measured using infrared beams. A virtual 24 × 24 cm square was considered the center zone. Data was compiled into five-minute blocks (Activity Monitor 7, Med Assoc., St. Albans, VT, USA). Due to a wrongful placement of the infrared beams on the apparatus, one subgroup of rats was excluded with regards to rearing activity during the first drug exposure.

### Elevated plus-maze

The EPM is a common method to assess both anxiety-like and disinhibited as well as explorative behavior (Carobrez and Bertoglio 2005; Ennaceur and Chazot 2016; Pellow et al. 1985). The EPM used in this study (Med Assoc., St. Albans, VT, USA) consisted of two open (10 × 50 × 1 cm) and two closed (10 × 50 × 40 cm) arms of black acrylic plastic, elevated one meter above the floor level, in an evenly lit space. Rats were allowed one hour of habituation to the room in their home cages on the day of the experiments and then individually for five minutes in a locomotor box, in order to stimulate exploratory behavior. Rats were placed in the center square, facing a corner of open/closed arms. Measurements were performed for five minutes, and entries into either open or closed arm, as well as time spent in either arm were analyzed.

### Field potential recordings

Ex vivo electrophysiological field potential recordings were performed following 48–96 h or 3 months of withdrawal, as previously described (Lagstrom et al. 2019). In brief, animals were briefly anesthetized with Isoflurane (Forene, Baxter, Apoteket AB, Sweden) before decapitation. The brain was quickly removed and coronal brain slices (300 µm) containing the striatum and the overlaying cortex were prepared using a Leica VT 1200S Vibratome (Leica Microsystems AB, Bromma, Sweden) in an ice cold modified artificial cerebrospinal fluid (aCSF), consisting of (in mM): 220 sucrose, 2 KCl, 6MgCl_2_, 26 NaHCO_3_, 1.3 NaH_2_PO_4_, 0.2 CaCl_2_ and 10 D-glucose. Slices were then kept in normal aCSF throughout the day, consisting of (in mM): 124 NaCl, 4.5 KCl, 2 CaCl_2_, 1 MgCl_2_, 26 NaHCO_3_, 1.2 NaH_2_PO_4_, and 10 d-glucose, with osmolarity adjusted to 315–320 mOsm with sucrose. Slices were allowed to equilibrate for 30 min at 30 °C and an additional 30 min at room temperature before starting recordings, and continuously oxygenated with 95% O_2_/5% CO_2_ at all times.

One hemisphere slice was placed in the recording chamber, with a constant flow of preheated aCSF (30 °C, 1.5 ml/min). A stimulating electrode (Monopolar tungsten electrode, TM33B, World Precision Instruments) and a recording electrode (prepared from borosilicate glass micropipettes with a Flaming/brown micropuller, Sutter Instruments, Novato, CA, Outer diameter 1.5 µm, resistance ranging from 2.5 to 4.5 MΩ) were placed in the brain area of interest. Electrodes were placed close to the overlying cortex for DLS, intrastriatal in DMS and nAc shell and core areas. Population spikes (PSs) were evoked using a paired-pulse stimulation protocol (50 ms interpulse interval, evoked with a frequency of 0.05 Hz). Stimulus/response curves were measured by stepwise increasing the stimulation intensity. Paired pulse ratio (PPR, PS2/PS1), considered as a measurement of release probability, was measured for three minutes at baseline and after drug perfusion. To investigate if putative neuroadaptations could be driven by changes in inhibitory transmission, the slices were treated with the GABA_A_ antagonist (-)-bicuculline methiodide (Tocris, Abingdon, UK) (dissolved in H_2_O to 20 mM stock solution, and further to 20 µM in aCSF prior to use). A PS amplitude of approximately 2/3 of the maximum PS amplitude was set for baseline, and a stable baseline was recorded for ten minutes before the drug was washed on. Signals were amplified with a custom-made amplifier, filtered at 3 kHz, and digitized at 8 kHz. Responses with a baseline PS amplitude of lower than 0.2 mV were excluded from analysis. For correlational analysis, a mean value of evoked PS amplitudes (at 24 μA) was calculated for each animal. Only animals with multiple recordings were included in the analysis.

### Statistical analysis

All data were considered with the presence of normal distribution, and parametric statistical tests were used. Possible outliers were tested for with Grubbs’ test, and positive outliers were excluded. When considering behavioral data, no rats showed deviant results in more than one of the performed measurements, and therefore no behavioral measurements were considered outliers. Behavioral data were analyzed using repeated measurement for analysis of variance (ANOVA) for analysis between treatment groups over time, paired t test for analysis within one group between separate measurement days, and unpaired t test for analysis between treatment groups. When analyzing all four groups together, one-way ANOVA together with Tukey's multiple comparisons test was used. A two-way ANOVA was used for data analysis of treatment effects over time during drug wash-on, as well as for the stimulus/response curve. Analysis of PPR was performed with an unpaired t test between treatment groups. Clampex 10.1 and (Molecular Devices, Foster City, CA, USA), Microsoft Excel (Redmond, WA), and Graph pad Prism 9 (GraphPad Software Inc., San Diego, CA, USA) were used for data analysis, and all data is presented as mean ± SEM, with the level of significance set to p < 0.05.

## Results

### Repeated nicotine administration sensitizes female rats to the locomotor stimulatory properties of nicotine

Female Wistar rats received 15 injections of either nicotine (0.36 mg/kg, subcutaneous) or saline in a discontinuous manner over 3 weeks, and locomotion together with rearing activity were measured following the first and the fifteenth injections (Fig. 1a). Repeated nicotine treatment did not significantly affect the weight of the rats during the treatment period (F_(1, 43)_ = 0.8718; p = 0.3557) or during protracted withdrawal for the subgroup of rats that continued into 3 months of withdrawal (2-way ANOVA: F_(1, 16)_ = 1.087; p = 0.3126) (data not shown).

Nicotine treatment increased locomotor activity both after the first and the fifteenth nicotine injection (2-way ANOVA. Nic 1st vs Veh 1st: F_(1, 43)_ = 17.31, p = 0.001; Nic 15th vs Veh 15th: F_(1, 43)_ = 36.33, p < 0.001) (Fig. 1b, c). 15 days of intermittent nicotine treatment produced a robust behavioral sensitization to the locomotor stimulatory effect of nicotine (paired t test. Nic 1st vs Nic 15th: t_22_ = 6.724, p < 0.001), with a slight increase also in the vehicle-treated group (Veh 1st vs Veh 15th: t_21_ = 2.303, p = 0.032) (Fig. 1d). Nicotine exposure further increased the time spent in the center zone, and repeated exposure further prolonged the duration (Dunnett´s multiple comparison test: Nic 1st vs. Veh 1st: t_22.23_ = 3.054, p = 0.0171; Nic 1st vs. Nic 15th: t_28.69_ = 2.701, p = 0.0335) (Fig. 1e). Locomotion during the initial five minutes of habituation to the test environment was not significantly affected by repeated nicotine exposure (Nic 15th vs. Veh 15th: t_43_ = 0.8341 p = 0.4088) (Fig. 1f). Rearing activity was not affected by nicotine in nicotine naïve rats, but an increased rearing was observed with repeated nicotine exposure (Nic 1st vs Veh 1st: F_(1, 31)_ = 0.010, p = 0.920; Nic 15th vs Veh 15th: F_(1, 43)_ = 18.38, p = 0.001; Nic 1st vs Nic 15th: t_16_ = 5.335, p < 0.001) (Fig. 1g, h). Rearing activity for vehicle treated rats did not change over time (Veh 1st vs Veh 15th: t_15_ = 1.036, p = 0.317) (Data not shown).

### No significant differences between treatment groups when assessed on the elevated plus-maze

**Fig. 2:**
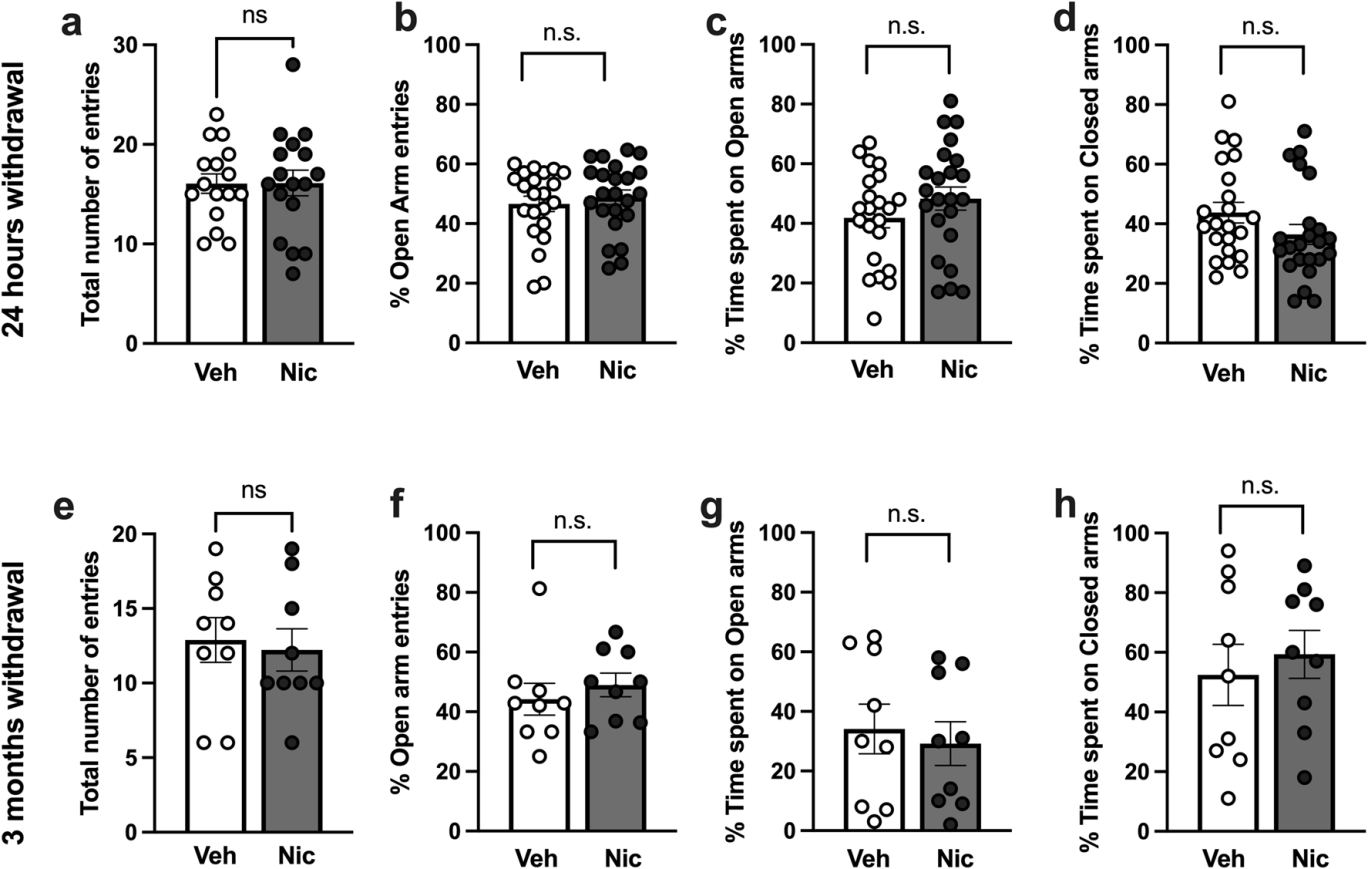
Behavior on the elevated plus-maze was not affected by repeated nicotine exposure followed by withdrawal. **a**–**d** No difference between treatment groups in the number of entries nor time spent in open and closed arms at the timepoint of 24 h of withdrawal. **e**–**h** Following three months withdrawal, rats previously receiving nicotine did not show an altered behavior on the EPM when compared to vehicle-treated control. Data are presented as mean ± SEM based on 22–23 rats (acute withdrawal) or nine rats (three months withdrawal) per group. *n.s.* non-significant effect by nicotine treatment on behavior as compared to controls

Repeated nicotine exposure followed by protracted abstinence has previously been shown to increase open arm duration on the EPM in male rats (Morud et al. 2018). To outline if a similar behavioral transformation would be present in female rats the animals were tested on the elevated plus-maze at the timepoint of 24 h of withdrawal from repeated passive nicotine administration, and a subgroup again at the timepoint of 3 months of withdrawal. When compared to vehicle-treated controls, 24 h of nicotine withdrawal did not affect the total number of entries into open and closed arms (t_(43)_ = 0.240, p = 0.811) or the percentage of entries into open arms (t_(43)_ = 0.626, p = 0.535) (Fig. 2a, b). The percentage of time spent in open (t_(43)_ = 1.239, p = 0.222) and closed (t_(43)_ = 1.527, p = 0.134) arms was also not altered by treatment (Fig. 2c, d). Following 3 months withdrawal, there were no significant differences with respect to total number of entries into open and closed arms (t_(16)_ = 0.322, p = 0.752), the percentage of entries into open arms (t_(16)_ = 0.723, p = 0.480), the percentage of time spent in open arms (t_(16)_ = 0.440, p = 0.666) or the percentage of time spent in closed arms (t_(16)_ = 0.530, p = 0.604) (Fig. 2e–h).

### Selective increase in accumbal neurotransmission during acute withdrawal from nicotine

**Fig. 3:**
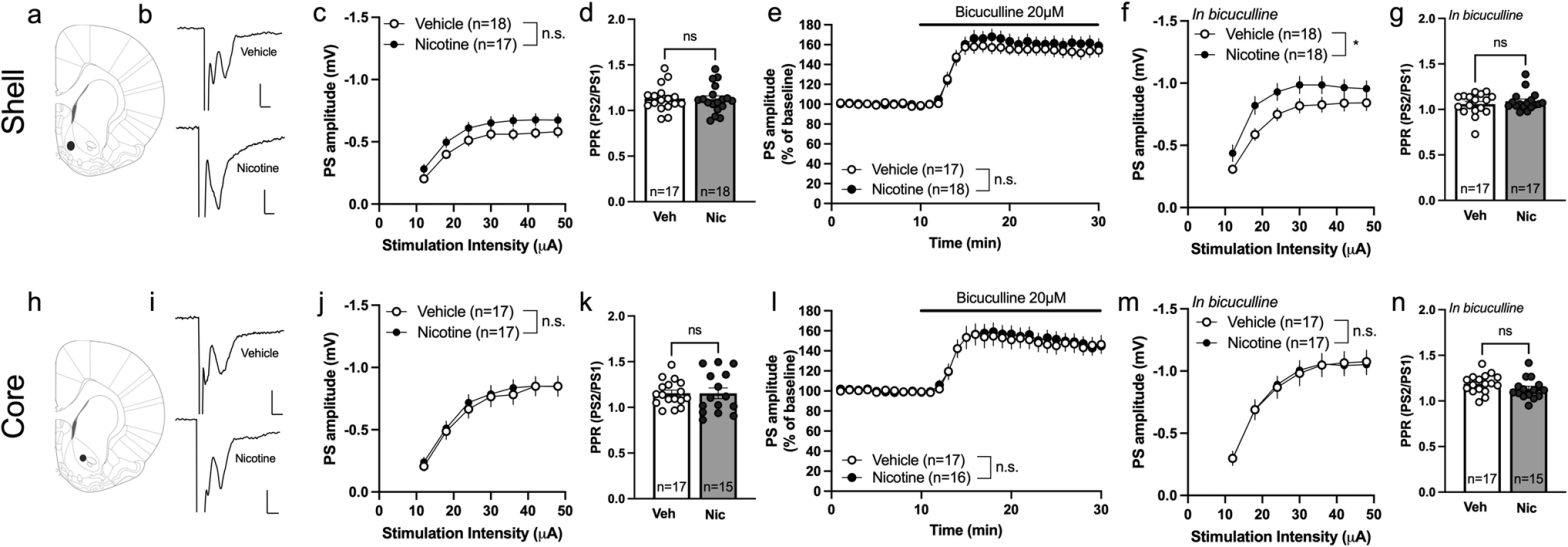
Selective increase in accumbal neurotransmission during acute withdrawal from nicotine. **a** A schematic drawing showing the region for placement of recording electrodes in nAc shell. **b** Example traces showing evoked PSs from vehicle and nicotine treated animals. Calibration 2 ms, 0.2 mV. **c** PS amplitudes evoked by a stepwise increase in stimulation intensity demonstrated a trend towards increased excitability in the nAc shell. **d** PPR was not affected by repeated nicotine treatment. **e** Wash on of bicuculline increased PS amplitudes in a similar manner in both treatment groups. **f**–**g** The stimulus/response curve following bicuculline wash on revealed an enhanced PS amplitude in slices from nicotine treated rats in nAc shell, without an effect on the PPR. **h** Schematic drawing showing the placement of electrodes in nAc core. **i** Example traces showing the recorded PS amplitudes from vehicle and nicotine treated animals. Calibration 2 ms, 0.2 mV. **j**–**n** In nAc core, previous nicotine treatment did not affect any of the measured parameters. Data are presented as mean ± SEM. *n* number of recordings, taken from 12 animals per group. *p < 0.05, significant effect by nicotine treatment

A subgroup of rats was taken into ex vivo field potential recordings 48–96 h after the last nicotine injection. Stepwise increasing the stimulation intensity showed no significant effect by treatment on evoked PS amplitude in either ventral striatal areas (2-way ANOVA: Shell: F_(1, 33)_ = 3.23, p = 0.081; Core: F_(1, 32)_ = 0.098, p = 0.756) (Fig. 3c, j). There were also no significant differences between treatment groups with respect to PPR (unpaired t-test. Shell: t_33_ = 0.126, p = 0.900. Core: t_30_ = 0.008, p = 0.994) (Fig. 3d, k). The GABA_A_ blocker bicuculline increased PS amplitudes in a manner that was independent on previous drug exposure in both subregions (2-way ANOVA. Shell: F_(1, 33)_ = 0.534, p = 0.470. Core: F_(1, 31)_ = 0.054, p = 0.818 (Fig. 3e, l). However, when isolating excitatory neurotransmission through bicuculline perfusion, a significant enhancement of stimulus/response curves were apparent in the nAc shell when comparing nicotine-treated rats with vehicle-treated controls (F_(1, 34)_ = 4.348, p = 0.045) (Fig. 3f). PPR was however still not affected (t_32_ = 0.794, p = 0.433) (Fig. 3g). Isolation of excitatory neurotransmission did not expose any effects by treatment in the nAc core (Stimulus/response curve: F_(1, 32)_ = 0.0002, p = 0.988. PPR: t_30_ = 1.466, p = 0.153) (Fig. 3m, n).

### Increased neurotransmission in DMS but not DLS during acute withdrawal from nicotine

**Fig. 4:**
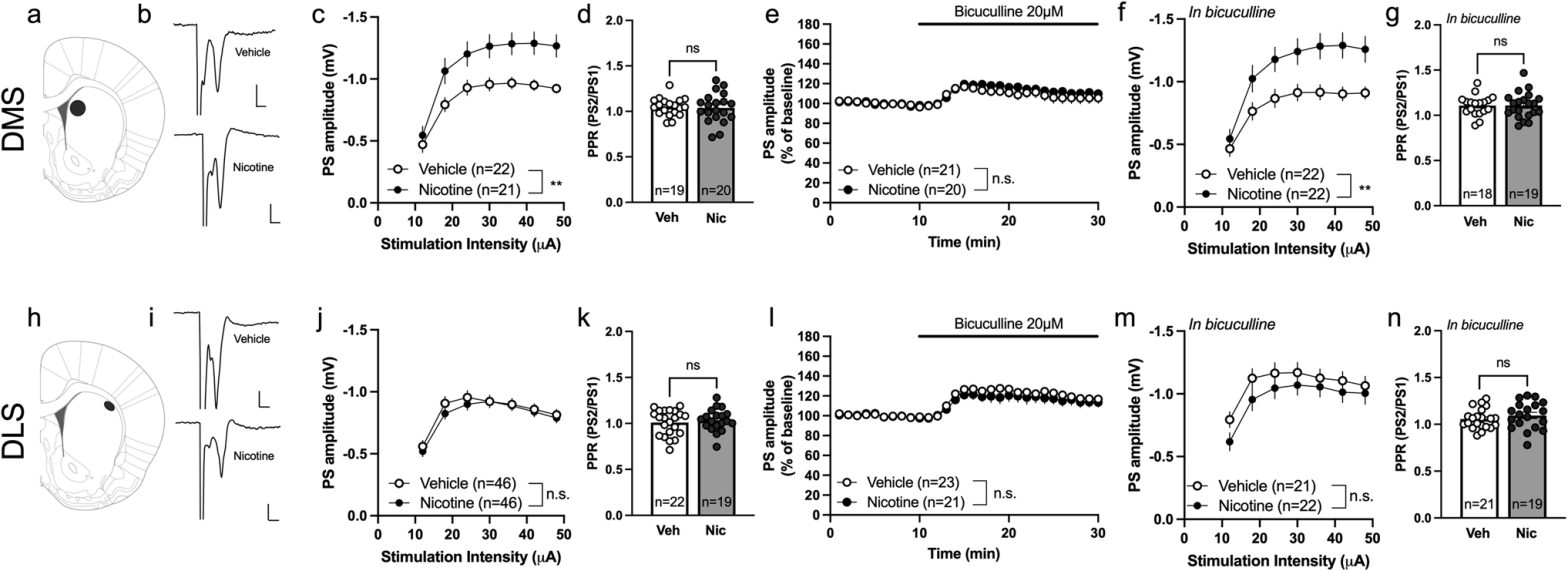
Increased neurotransmission in DMS but not in DLS during acute withdrawal from nicotine. **a** Schematic drawing showing the placement of electrodes in dorsomedial striatum. **b** Example traces showing evoked PS amplitudes from vehicle and nicotine treated animals. Calibration 2 ms, 0.2 mV. **c**–**d** Stimulus/response curves revealed that evoked PS amplitudes were significantly enhanced in the DMS of nicotine treated animals, without affecting PPR. **e** Bicuculline wash on elevated PS amplitudes in a similar manner in both treatment groups. **f** The increase in PS amplitude observed in brain slices from nicotine exposed rats sustained during bicuculline exposure. **g** PPR was not affected by treatment. **h** Schematic drawing showing the placement of electrodes in dorsolateral striatum. **i** Example traces showing evoked PS amplitudes from vehicle and nicotine treated animals. Calibration 2 ms, 0.2 mV. **j**–**n** No differences between treatment groups were observed in DLS 48–96 h into nicotine withdrawal. Data are presented as mean ± SEM. *n* number of recordings, taken from 12 animals per group. *p < 0.05, significant effect by nicotine treatment

Recordings performed in the dorsal striatum during acute withdrawal demonstrated a potentiation of stimulus/repose curves in the DMS from rats previously receiving nicotine (2-way ANOVA: F_(1, 41)_ = 9.270, p = 0.004), with no concomitant effect on PPR (unpaired t test. t_37_ = 0.227, p = 0.822) (Fig. 4c, d). Wash on of the GABA_A_ antagonist bicuculline increased PS amplitudes in a similar manner in both nicotine treated and vehicle animals (2-way ANOVA. F_(1, 42)_ = 2.028, p = 0.162), and the increase in stimulus/response curve amplitude sustained indicating that effects are connected to increased excitatory neurotransmission (F_(1, 42)_ = 8.123, p = 0.007) (Fig. 4e, f). No treatment effect on PPR was observed following bicuculline exposure (t_37_ = 0.019, p = 0.985) (Fig. 4g). Nicotine-treatment did not significantly affect stimulus/response curves or PPR in DLS when measured 48–96 h into withdrawal (stimulus/response curve: F_(1, 90)_ = 0.366, p = 0.547; PPR: t_39_ = 0.635, p = 0.529) (Fig. 4j, k). Bicuculline disinhibited neurotransmission to a similar extent independent on treatment group (Fig. 4l). While a trend towards depressed PS amplitude was observed at lower intensities following bicuculline treatment, there was no significant effect by treatment on stimulus response curves (F_(1, 42)_ = 0.692, p = 0.410) (Fig. 4m). No effect by treatment was observed with regards to PPR during bicuculline perfusion (t_38_ = 0.929, p = 0.359) (Fig. 4n).

### Evoked neurotransmission in the nucleus accumbens correlates with nicotine-induced locomotor activity

**Fig. 5:**
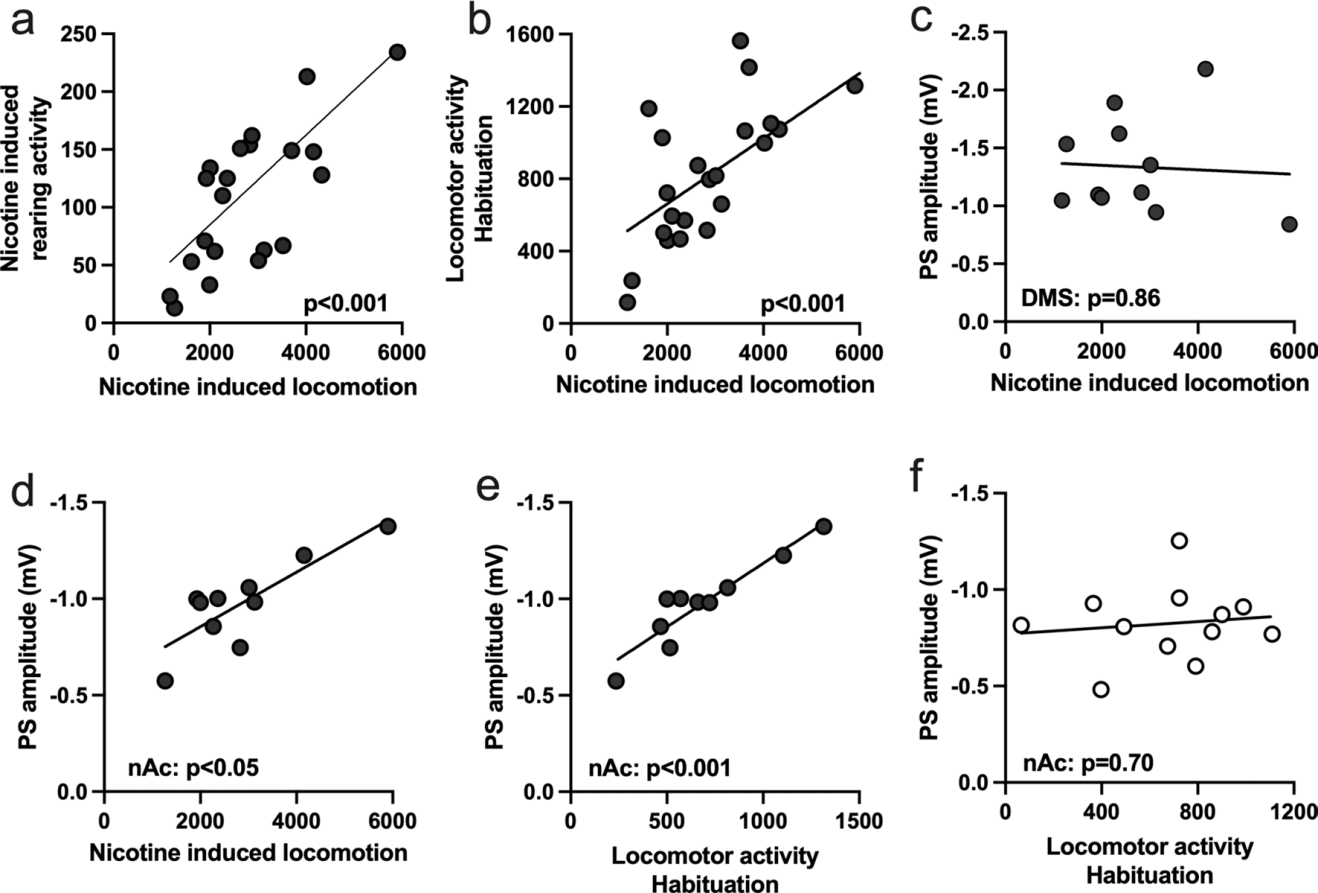
Evoked neurotransmission in the nucleus accumbens correlates with nicotine-induced locomotor activity. **a**–**b** Nicotine-induced locomotion in individual animals correlated with both nicotine induced rearing activity as well as locomotion during habituation for that same animal. **c** Evoked PS amplitudes in the DMS did not correlate with nicotine-induced locomotion. **d**–**e** Evoked PS amplitude in nAc shell correlated with both nicotine induced locomotion and locomotion during the habituation phase. **f** Recordings performed in brain slices from vehicle-treated rats did not correlate with locomotor activity during habituation. Data are comparing mean values from individual animals (n = 23–10/group)

In a way to outline putative associations between striatal neuroadaptations and behavioral output, data retrieved from individual animals were compared using simple linear regression. Nicotine induced locomotion in individual animals correlated with both nicotine induced rearing activity (Simple linear regression: F_(1, 21)_ = 28.36, p < 0.001; R^2^ = 0.57) (Fig. 5a), and baseline locomotor activity during the first five minutes of habituation (F_(1, 21)_ = 24.83, p < 0.001; R^2^ = 0.54) (Fig. 5b), but not with the time spent on open arms (F_(1, 21)_ = 0.0048, p = 0.9455, R^2^ = 0.0002) (data not shown). Electrophysiological recordings performed on bicuculline-treated brain slices from nicotine exposed rat demonstrated no correlation between evoked PS amplitude in the DMS and nicotine-induced locomotion (F_(1, 9)_ = 0.0345, p = 0.8566; R^2^ = 0.0038) (Fig. 5c). However, evoked responses in the nAc shell correlated with both nicotine-induced locomotion (F_(1, 8)_ = 17.78, p = 0.0029; R^2^ = 0.690) (Fig. 5d), and locomotion during habituation (F_(1, 8)_ = 54.2, p < 0.001; R^2^ = 0.871) (Fig. 5e). Interestingly, evoked responses from vehicle treated rats did not correlate with locomotion during the first five minutes of habituation (F_(1, 10)_ = 0.1600, p = 0.6976; R^2^ = 0.0158) (Fig. 5f).

### The nicotine-induced changes in neurotransmission in striatum do not sustain following prolonged nicotine withdrawal

**Fig. 6:**
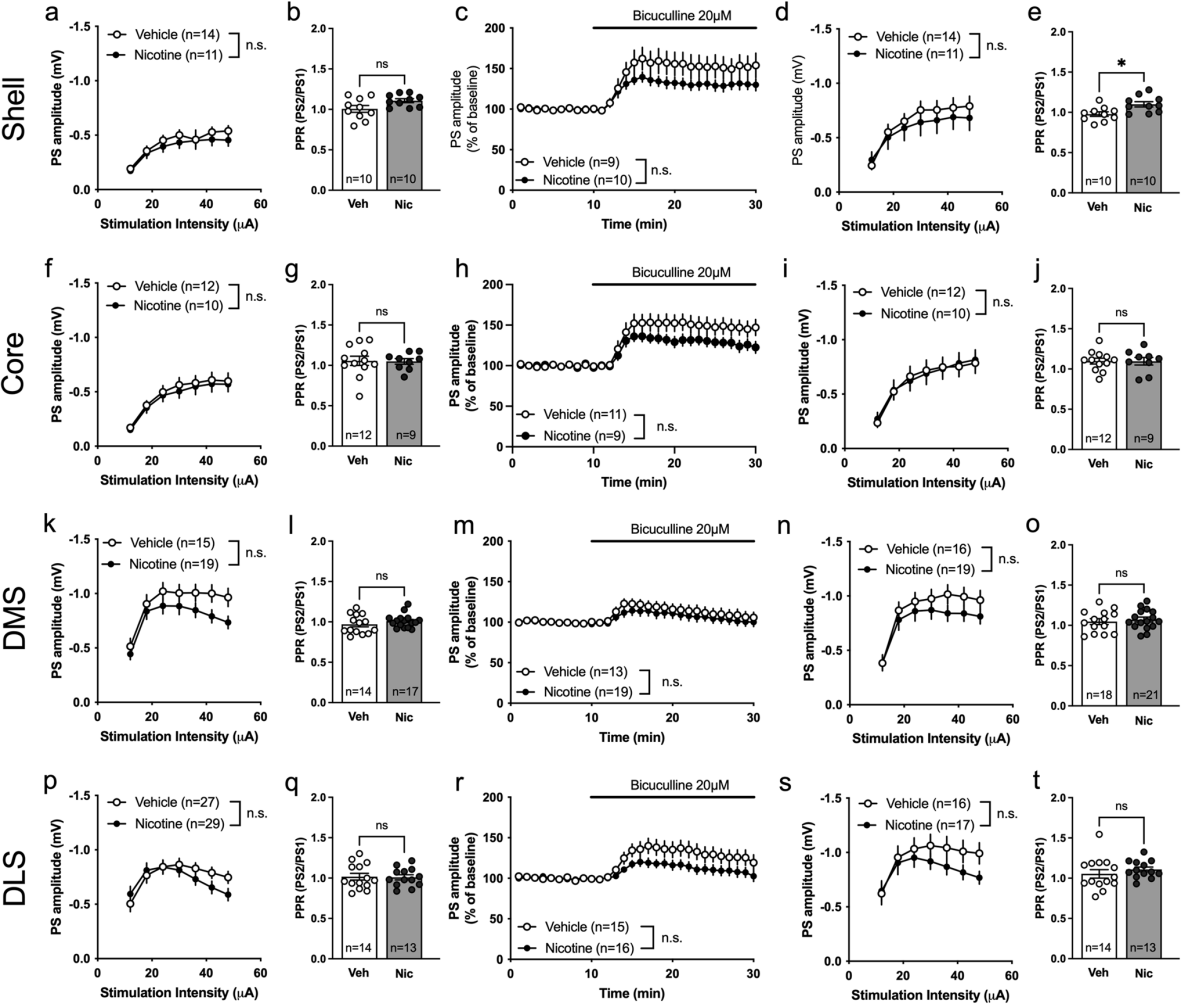
Nicotine-induced changes in neurotransmission in striatum does not sustain following prolonged nicotine withdrawal. **a**, **d** In the nAc shell, stimulus/response curves were not affected by previous nicotine treatment. **b**, **e** PPR measured in aCSF demonstrated a trend towards reduced probability of transmitter release, and this effect was further established in bicuculline-treated slices. **c** While a visual trend towards a reduced GABAergic tone was observed during bicuculline-perfusion, the effect was not significant. **f**–**j** None of the measurements performed demonstrated an effect by treatment in nAc core. **k**–**o** The increase in excitability observed during acute withdrawal was no longer present in the DMS following extended withdrawal. **p**–**t** While there was a trend towards reduced disinhibition during bicuculline perfusion, none of the parameters measured were significantly modulated in the DLS. Data are presented as mean ± SEM. *n* number of recordings, taken from 9 animals per group. *p < 0.05, significant effect by nicotine treatment

Progressive neuroadaptations that develop during prolonged withdrawal have previously been reported in male rats (Morud et al. 2018). To determine if changes in neurotransmission were long-lasting, electrophysiological recordings were performed after 3 months of withdrawal. After 3 months withdrawal, there were no significant effects by treatment on stimulus/response curves or PPR in the nAc shell (Stimulus/response: F_(1, 23)_ = 0.537, p = 0.471; PPR: t_18_ = 2.087, p = 0.051) (Fig. 6a, b). Although there was no significant difference in the increase of PS amplitude elicited by bicuculline (F_(1, 17)_ = 2.290, p = 0.149), or stimulus/response curves after bicuculline exposure (F_(1, 23)_ = 0.299, p = 0.590), PPR was increased in rats previously receiving nicotine treatment (t_18_ = 2.840, p = 0.0109) (Fig. 6c–e). While a trend towards reduced GABAergic tone was indicated in several brain subregions, there were no significant effects on any parameter in any other brain region tested. Neither the nAc core (stimulus/response curve: F_(1, 20)_ = 0.192, p = 0.666; PPR: t_19_ = 0.097, p = 0.924; Bicuculline wash on: F_(1, 18)_ = 2.429, p = 0.137; Stimulus/response curve in bicuculline: F_(1, 20)_ = 0.001, p = 0.977; PPR in bicuculline: t_19_ = 0.030, p = 0.977) (Fig. 6f–j), the DMS (Stimulus/response curve: F_(1, 32)_ = 2.578, p = 0.118. PPR: t_28_ = 1.077, p = 0.291; Bicuculline wash on: F_(1, 30)_ = 0.526, p = 0.474; Stimulus/response curve in bicuculline: F_(1, 33)_ = 1.132, p = 0.295; PPR in bicuculline: t_28_ = 0.543, p = 0.591) (Fig. 6k–o), nor the DLS (stimulus/response curve: F_(1, 54)_ = 1.054, p = 0.309; PPR: t_25_ = 0.140, p = 0.890; Bicuculline wash on: F_(1, 29)_ = 3.164, p = 0.086; Stimulus/response curve in bicuculline: F_(1, 31)_ = 1.075, p = 0.308; PPR in bicuculline: t_25_ = 0.889, p = 0.382) (Fig. 6p–t), were significantly altered compared to vehicle-treated control.

## Discussion

The data presented here suggest that 3 weeks of passive nicotine administration produces behavioral sensitization towards the locomotor stimulatory effects by nicotine in female rats but does not affect behavior on the EPM during acute or protracted withdrawal. In addition, electrophysiological recordings performed in striatal subregions during acute and protracted nicotine withdrawal suggest that nicotine increases excitatory neurotransmission in a subregion-specific manner. The increased amplitude of evoked response observed in nAc shell and DMS did not sustain after 3 months withdrawal. Instead, an overall reduction in GABAergic tone appeared to be present in several brain areas following extended withdrawal. Thus, while behavioral sensitization is robustly induced by nicotine in both male and female rats, long-lasting effects by nicotine with regards to behavior on the EPM, as well as the spatial sequence of striatal neuroadaptations, may occur in a partially sex-dependent manner.

Rats received discontinuous vehicle or nicotine injections for 15 days over 3 weeks and a robust sensitization towards the locomotor stimulatory properties of nicotine was seen consistently in all nicotine‐treated animals. Although a slight increase in locomotor activity also was detected in vehicle treated animals over time, the data presented here supports previous studies showing nicotine-induced behavioral sensitization of locomotor activity in female rats (Ericson et al. 2010; Lucente et al. 2022). Nicotine further increased rearing activity following repeated exposure, but not in nicotine in naïve rats. Possibly, acutely administered nicotine produces aversive effects that conceal the rearing-inducing effect that later is disclosed following tolerance development to the aversive properties. Alternatively, behavioral sensitization to subthreshold rearing-inducing effects of nicotine occurred regardless of acute aversive effects being present or not. It should be noted that nicotine naïve male rats in general demonstrate decreased rearing activity in response to nicotine (Adermark et al. 2015). One underlying factor why this was not observed in female rats could be associated with estrogen, which may affect nitric oxide signaling (El-Lakany et al. 2020; el-Mas et al. 2011). In the extension, a reduced aversive experience to nicotine could contribute to the finding that female rats are more sensitive to the acute rewarding effects of nicotine (Xue et al. 2020).

Repeated exposure to nicotine significantly increased the time spent in the center zone of the locomotor box indicating that nicotine exposure to sensitized animals may produce anxiolytic effects. Still, nicotine-treated rats did neither spend more time in open arms nor display an increase in the number of entries into open arms, suggesting no sustained effects by repeated nicotine on anxiolytic-like behavior. Considering that EPM measurements were conducted 24 h after the last nicotine injection, these effects may be counterbalanced by acute withdrawal effects. Importantly, studies of male Wistar rats have demonstrated that anxiolytic-like behavior on the EPM increases with protracted withdrawal, and that these behavioral transformations may be attributed to reduced neurotransmission in the nAc (Morud et al. 2018). Similar findings were not observed here, neither with respect to increased open arm duration nor synaptic depression in the nAc shell, indicating that these effects may be sex specific. However, even though there was a large interindividual variability, female control rats were exploratory active and did not demonstrate anxiogenic behavior with respect to the time spent in open arms. This finding is in line with previous studies, demonstrating that female rats have a higher baseline activity, and demonstrate a reduced responsiveness to the effects of anxiolytic drugs on the EPM when compared to males (Simpson et al. 2012). The EPM might thus be considered a less validated model for anxiolytic-like behavior in female rats.

In a way to outline striatal neuroadaptations elicited by repeated nicotine exposure electrophysiological field potential recordings were performed in striatal brain areas during acute and protracted withdrawal from nicotine. At the timepoint of acute withdrawal an increase in excitability was seen in both nAc shell and DMS in nicotine treated animals. In nAc shell, an increase in PS amplitude was only seen following isolation of glutamatergic neurotransmission by bath perfusion of bicuculline. In the DMS, which also demonstrated a lower GABAergic tone over evoked PS amplitudes, a robust increase of PS amplitude was observed in both aCSF exposed and bicuculline-treated slices. The lack of effect on GABAergic neurotransmission is partially in disagreement with findings from male rats (Licheri et al. 2020), suggesting that the previously observed increase in GABAergic neurotransmission during acute withdrawal may be sex specific. While GABAergic neurotransmission produces a tonic inhibition of striatal field potentials, measured responses primarily reflect glutamatergic neurotransmission, and evoked PS amplitudes are rapidly blocked by the AMPA receptor antagonist CNQX (Lagstrom et al. 2019; Domi et al. 2023a). Considering that PPR was not significantly modulated by treatment it is thus likely that the increased excitability is associated with changes in AMPA receptor subunit composition or increased AMPA receptor density, thereby strengthening AMPA receptor signaling. Importantly, increased excitatory neurotransmission and AMPA receptor expression has previously been demonstrated in male rats following nicotine self-administration (Domi et al. 2023b; Gipson et al. 2013), and may thus be an important neurobiological underpinning of nicotine addiction (Ruda-Kucerova et al. 2021).

The increased excitability observed in nAc shell and DMS did not sustain following 3 months of withdrawal, suggesting that changes in AMPA receptor expression/function are not long-lasting. In fact, at this time-point, there was instead a trend towards a depression of PS amplitude, at least during higher stimulation intensities. There was also a trend towards a reduced GABAergic tone in several striatal subregions. Reduced GABAergic tone following extended nicotine withdrawal is in agreement with previous studies performed in male rats. In fact, 3 months of nicotine withdrawal has been shown to both decrease GABAergic tone and to impair GABA_A_ receptor signaling (Morud et al. 2018). One underlying mechanism could be attributed to a long-lasting impairment of the K^+^–Cl^−^ cotransporter isoform 2 (KCC2), resulting in reduced chloride extrusion and shifts in GABA_A_ reversal potentials (Ostroumov et al. 2020; Thomas et al. 2018). Thus, while the temporal and spatial sequence of displayed neuroadaptations seems to develop in a sex-specific manner, protracted nicotine withdrawal still appears to display a similar influence over GABAergic neurotransmission.

While the role of displayed neuroadaptations for driving nicotine-induced behavioral transformations remains to be established, we found a correlation between evoked PS amplitude during acute withdrawal and nicotine-induced locomotion and locomotion during habituation. This is in agreement with the finding that chemogenetic activation of dopamine neurons projecting to the nAc, but not dopaminergic neurons in the substantia nigra, induces hyperactivity (Boekhoudt et al. 2016). Importantly, these associations were only present in rats receiving nicotine, supporting the idea that accumbal neuroadaptations and aberrant dopamine signaling are important neurobiological underpinnings of drug-induced behavioral sensitization (van Zessen et al. 2021; Jiang et al. 2021; Fennell et al. 2020; Robinson et al. 1988). However, more research is required to further establish these associations.

In conclusion, the data presented here demonstrates that nicotine produces robust behavioral sensitization in female rats and promotes sustained neuroplasticity in the nAc shell and DMS. These neuroadaptations are most likely associated with increased AMPA receptor signaling and do not sustain after 3 months withdrawal. Instead, a trend towards a hypoGABAergic state is indicated after protracted withdrawal, which is also supported by studies performed on male rats. Overall, while some behavioral and neurophysiological transformations elicited by nicotine appear to be similar, the role of sex should be considered when performing studies of nicotine pharmacology and, in the extension, addiction.

## Data Availability

The data presented in this manuscript can be made available on reasonable request.
